# Extensive Subcutaneous Rheumatoid Nodules Reflecting High Burden of Disease Activity

**DOI:** 10.31138/mjr.200625.rha

**Published:** 2026-04-21

**Authors:** Georgios A. Drosos, Anastasia K. Zikou, Paraskevi V. Voulgari, Alexandros A. Drosos

**Affiliations:** 1 Department of Rheumatology, School of Health Sciences, Faculty of Medicine, University of Ioannina, Ioannina, Greece;; 2 Department of Radiology, Medical School, University of Ioannina, Ioannina, Greece

**Keywords:** arthritis, rheumatoid, rheumatoid nodule, subcutaneous nodules, female, methotrexate

## Abstract

Rheumatoid arthritis (RA) is a chronic autoimmune systemic disease affecting mostly the small joints of the hands and feet. Extra-articular manifestations (EAMs) comprise the involvement of the skin, eyes, heart and lung. Among them, subcutaneous rheumatoid nodules (RN) are a common manifestation of systemic disease. However, disorders such as rheumatoid nodulosis, multicentric reticulohistocytosis (MRH) and others present subcutaneous nodules, similar to those seen in RA patients. A 41-year-old woman with a 10-year history of untreated RA, presented complaining of pain and tenderness in the small joints of the hands and feet. Past medical and family history were unremarkable. She presented swelling and tenderness on all metacarpophalangeal joints (MCPs) bilaterally and had multiple widespread subcutaneous RN affecting almost all the fingers of the hands, as well as, the Achille’s tendons in a symmetrical manner. She had elevated levels of acute phase reactants and high titres of autoantibodies. Hand x-rays revealed severe erosive changes affecting all MCPs joints bilaterally. Thus, in this review we discuss the differential diagnosis of subcutaneous RN and their role in RA disease activity. This is an educational case of a patient with RA and extensive subcutaneous RN affecting the upper and lower extremities. To this end, physicians must be familiar and recognise early these signs which reflect RA disease activity and treat appropriately.

## INTRODUCTION

Rheumatoid arthritis (RA) is a chronic autoimmune systemic disease that predominantly affects the synovial membrane of the joints, leading to joint damage and bone destruction.^1^ Extra-articular manifestations (EAM) include rheumatoid nodules (RN) as well as the involvement of the lung, heart, eyes and other organs.^1^ Therefore, RN are an indicator of RA disease activity level and severity. However, many disorders such as, rheumatoid nodulosis,^2^ and multicentric reticulohistocytosis (MRH)^3^ present multiple subcutaneous nodules similar to those seen in RA patients.^4,5^ Rheumatoid nodulosis is a term used to describe multiple widespread subcutaneous RN, which is related to the treatment of RA with conventional synthetic disease modifying anti-rheumatic drugs (csDMARDs), especially methotrexate (MTX), or biological (b) DMARDs^6,7^ while, MRH is a disorder related to malignancies.^3^ Thus in this review we present a RA patient with extensive subcutaneous RN and we discuss the differential diagnosis and the role of subcutaneous RN on disease burden. Written informed consent was obtained from the patient for publication of this case report and any accompanying images. Ethics approval obtained for this manuscript by the scientific board of Hospital University of Ioannina.

## CASE PRESENTATION

**Figure 1.**
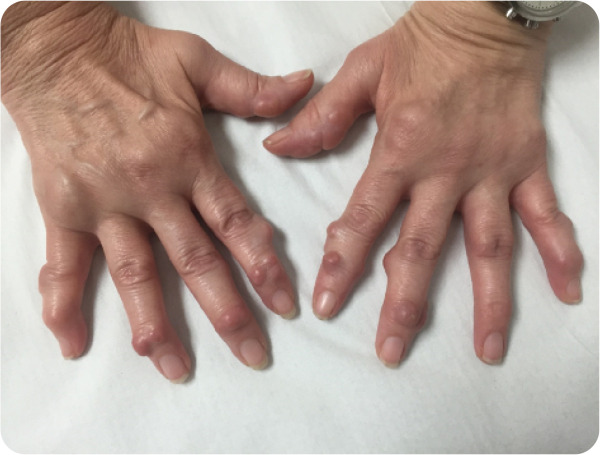
Multiple subcutaneous rheumatoid nodules in various sizes affecting almost all digits of both hands. Note also the swelling of all metacarpophalangeal joints as well as muscle atrophy in both hands.

**Figure 2.**
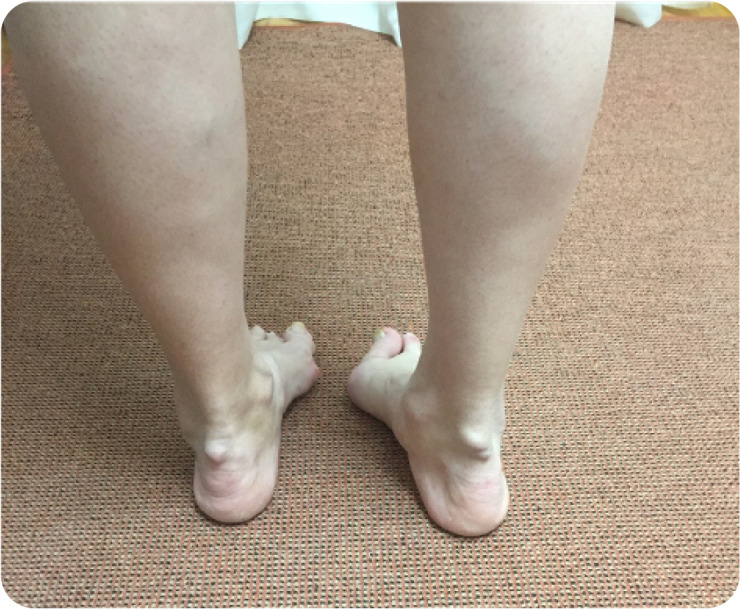
Subcutaneous rheumatoid nodules involving the Achilles tendons bilaterally. Subluxations and hallux valgus of the first metatarsophalangeal joints are shown bilaterally.

**Figure 3.**
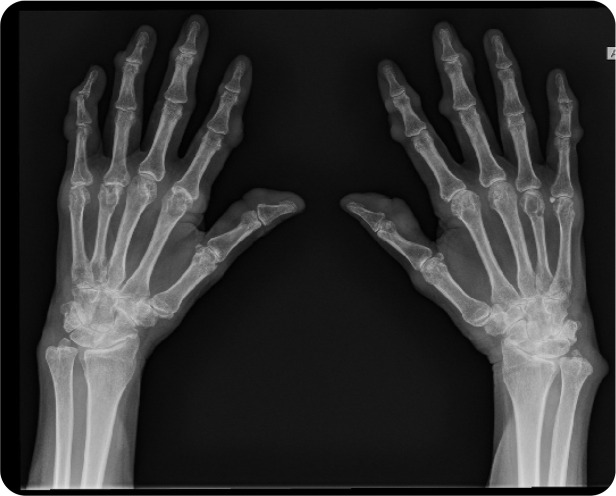
Hand and wrist x-rays of the same patient. Asymmetrical soft tissue swelling is evident in almost all digits, reflecting the presence of subcutaneous nodules. Severe destructive changes affecting all metacarpophalangeal joints and joint space narrowing are depicted bilaterally. Joint space narrowing and cyst formation cited in carpal bones. Finally, erosive changes are present in the right hand affecting the 3rd and the 5th distal interphalangeal joints.

A 41-year-old woman with a 10-year history of RA, presented to us with swelling and tenderness of the small joints of the hands. Past medical and family history were unremarkable. She was no smoker. She wished to get pregnant and denied any treatment, except for small doses of prednisone (5–7,5mg/day) and/or nonsteroid anti-inflammatory drugs. Physical examination revealed swelling and tenderness of the metacarpophalangeal joints (MCPs), atrophy of the interosseous muscles of the hands and multiple subcutaneous RN on the fingers of the hands (**Figure 1**) and under the Achille’s tendon bilaterally (**Figure 2**). Laboratory evaluation revealed high C-reactive protein (C-RP) 60mg/L (normal value<6), erythrocyte sedimentation rate (ESR) 81mm/h, positive rheumatoid factor (RF) 205 U (normal value <15U), positive anticitrullinated protein antibodies (ACPA) 244U (normal value<10U). The rest of immunological and laboratory tests were within normal limits. Hand and wrist x-rays showed severe erosive changes and joint space narrowing affecting almost all MCPs, as well as the carpal bones (**Figure 3**). Chest x-ray was normal. Our patient was treated with adalimumab (ADA) 40mg every 2 weeks subcutaneously, along with 10mg of prednisone per day. After 3 months of treatment, a substantial clinical and laboratory response was noted with normalisation of acute phase reactants; however, the features of RN did not change. The dose of prednisone was tapered and she continued receiving ADA.

## DISCUSSION

Subcutaneous RN occur in about 10,5% of RA patients,^8,9^ however they may also affect, but less frequently, other parts of the body such as the sclera of the eyes, lung parenchyma,^10,11^ vocal cords, heart valves, and others. Subcutaneous RN are typically distributed over the areas of repeated trauma or pressure and occur adjacent to the joints on extensor surfaces, such as the elbows, forearms, fingers and Achilles’ tendons. Subcutaneous RN are well demarcated flash-coloured lumps or masses, which are movable and frequently adherent to underline periosteum. They vary in size from small to pea size lesions, or large such as the size of a small lemon. Upon palpation, subcutaneous RN may feel either doughy or firm, are usually not tender unless there is inflammation, ulceration or infection. They manifest mostly in male RA patients, smokers and in those with high titres of RF or/and ACPA.^12^ Subcutaneous RN biopsy shows a typical feature of central necrosis, surrounded by palisading histiocytes and macrophages with an out layer of fibroblasts, lymphocytes and plasma cells.^13^

Subcutaneous RN present diagnostic challenges as they can resemble other subcutaneous lesions such as tophaceous lesions, cholesterol xanthomas, epidermal cysts, subcutaneous granuloma annulare, metastatic lesions, basal cell carcinomas, lupus panniculitis, sarcoidosis and rheumatic fever subcutaneous nodules.^4,5^ Our patient had no hyperuricemia and gout arthritis, neither high cholesterol levels, she had no history of rheumatic fever nor lupus, neither sarcoidosis. Other conditions which must be excluded are MRH and rheumatoid nodulosis related to csDMARDs treatment, especially MTX. MRH is a rare disorder affecting mostly women older than 50 years of age. In these cases, patients present arthralgias and arthritis affecting the hands, with papulonodular skin lesions involving mostly periungual and dorsal hands as well as, mucosal nodulosis. It is associated with malignancy (melanoma, endometrial, peritoneal and lung carcinoma). Hand x-rays reveal erosive changes affecting the distal inter-phalangeal joints (DIPs), a picture similar to arthritis mutilans.^6,7^ The present case is a young woman with an established RA with clinical, laboratory and imaging findings different from those seen in MRH patients. Our patient had also limited DIPs joints involvement, which may be seen in some severe cases of RA, as the patient we presented. On the other hand, DIPs involvement is a characteristic picture of osteoarthritis, psoriatic arthritis (PsA) mutilans, as well as MRH. In the last disorder the radiographic changes differ from those seen in RA.^14^

One uncommon adverse event of MTX therapy is the accelerated rheumatoid nodulosis. It is characterised by the rapid development of subcutaneous nodules resembling RN. Rheumatoid nodulosis presents as painful or tender nodules affecting mostly the subcutaneous tissues of the hands and feet. These nodules are numerous and small, appear away from the joints and the estimated incidence is 8%.^15^ However, Patatanian et al., in a review study of MTX induced accelerated nodulosis, reported that out of the 27 cases listed, only 4 of them satisfied the Naranjo scale for documentation of MTX induced such adverse events. ^16^ It is of interest to note that the Naranjo scale is a method for estimating the probability of adverse drug reactions. ^17^ Other drugs reported to be associated with rheumatoid nodulosis are: leflunomide (LFN), azathioprine (AZA) as well as etanercept (ETN), infliximab (IFX), tocilizumab (TCZ), and others. The cumulative dose of MTX associated with rheumatoid nodulosis development ranges from 90 to 7200mg and it can occur between 3 months to 12 years after the initiation of MTX therapy.^2^ In these cases RA disease activity is usually low without elevation of acute phase reactants, while RF or/and ACPA could be positive. Indeed, a literature review by Palmeiro et al. retrieved a total of 109 cases of accelerated nodulosis from 39 studies. The mean age was 52.7 years, 65% were women, and most of the patients had RA 99/109 (90.8%). Other diagnosis included juvenile idiopathic arthritis (N:4), (PsA) (N:3), systemic lupus erythematosus (N:1), dermatomyositis (N:1) and aortitis (N:1). As regards RA patients, out of 84 that were tested, 67 (79.8%) had positive RF. Concerning the offended agent, MTX was the most frequent 81.2%, followed by ETN (6.4%), TCZ (5.5%), LFN (2.8%), AZA (1.8%), and IFX (0.9%). On histological level, drug induced accelerated nodulosis has similar features to RA- related RN. Concerning treatment, the major part of patients discontinued the causative agent and were treated mostly with steroids, colchicine, HCQ, SSZ, and D-penicillamine or cyclophosphamide.^18^ Furthermore, Subramanian et al. recently reported a case series of five patients with seropositive RA treated with MTX who developed accelerated nodulosis despite that they responded to MTX treatment. Four patients discontinued MTX, while one continued MTX therapy. Two patients received hydroxychloroquine (HCQ) one LFN and one sulfasalazine (SSZ). They concluded that there is a need for vigilance in RA patients treated with MTX.^2^ The mechanism of MTX induced rheumatoid nodulosis is unclear. MTX induces adenosine production from inflammatory infiltrated monocytes activating adenosine A1 receptors and enhancing giant cell formation, in vitro.^19^ Our patient had never received any treatment with csDMARDs or/and bDMARDs, thus rheumatoid nodulosis is excluded.

Our patient had a chronic untreated RA, with high acute phase reactants and high levels of autoantibodies along with severe destructive changes as depicted in the hand x-ray. Therefore, subcutaneous RN in our patient is related to the disease process and reflect the high burden of disease activity. Biopsy was not performed as the diagnosis was obvious. To prevent the development of RN in RA is difficult, but proper and early management of the disease could reduce the rate of RN formation.^20^ Additionally, advice on smoking cessation, increase of physical activity, and a healthy diet plan are mandatory. The management of rheumatoid nodulosis is challenging. MTX discontinuation is the first step and additional treatments are the use of colchicine, HCQ, or SSZ, along with the use of steroids. In some cases rheumatoid nodules regress spontaneously.^2,18^ This case illustrates that seropositive RA is associated with subcutaneous RN, reflecting a more severe and destructive disease. Consequently, early intervention with tight control treatment,^20^ close follow-up and monitoring are necessary.

## CONCLUSIONS

Subcutaneous RN are common EAM of RA reflecting severe disease. They can also be found in many other disorders, including autoimmune rheumatic diseases like systemic lupus erythematosus, rheumatic fever, sarcoidosis, and metabolic diseases such as dyslipidaemia and gout. Furthermore, rheumatoid nodulosis could appear in RA patients treated with MTX. Finally, similar lesions are also present in patients with MRH, a disorder associated with malignancies. The diagnosis of subcutaneous RN remains challenging, is usually based on clinical findings and histopathological examination when needed. However, the presence of subcutaneous RN may be a marker of EAM of RA and a predictor of severe disease, as in our patient. Thus, recognition of subcutaneous RN, early treatment and control of disease activity are mandatory.
